# Miscellaneous

**Published:** 1887-01

**Authors:** 


### MISCELLANEOUS.

"YOUNG man," said a revivalist solemnly, "do you feel that you are prepared to answer the summons at any moment? Do you realize that when you go to bed at night you may be called before the morning dawns?"

"Oh, yes, sir. I'm night clerk in a drug store, an' all you've got to do is to keep on ringin' the bell until you hear me holler."

LAW AND MEDICINE.—It often happens that "smart" young limbs of the law take great delight in asking superfluous questions of medical witnesses, when on the stand, with a view to puzzling or annoying them. One such was cross-examining the celebrated French chemist Orfila, and put him the question whether he could state the precise amount of arsenic requisite to kill a fly. "Certainly," replied the expert; "but I must know beforehand the age of the fly, its sex, its temperament, its condition, and habits of body, whether married or single, widow or maiden, widower or bachelor."

A QUEER CASE.—*La Revista Clinica* of June, 1885, reports the following rare case: An alcoholist, 66 years of age, was admitted into the hospital of Bologna for the treatment of scurvy. After his recovery from this affection, it was found that, in spite of the greatest efforts, he could only walk backward, and that after he had to turn around his own axis. These movements, of course, constantly imperilled his life. He soon succumbed to pneumonia, and the examination of the brain showed an atheromatous condition of the vessels at the base. We wonder that the inspection of the spinal cord was omitted, which might have given some clue for these peculiar symptoms.—*Ibid.*

EPIDEMIC DIPHTHERIA.—The Columbus, O., Board of Education committee on hygiene, in the matter of diphtheria epidemic prevailing in the Spring-street school district, believes that it has traced the disease to its origin, and that it is located in a double brick house in an alley between Eigthth and Ninth streets. There are unmistakable evidences that the dread disease was propagated there, as the cellar has for some time been foul with the germs of such diseases. The water which the people use for drinking also comes from a well but eight feet from a vault, while other wells are close to the place.

FATAL PLUMBING.—Imperfect and leaky plumbing caused the death, by diphtheria, recently of two children in a family in St. Paul, Minn. So the doctors said who attended the cases and tested the work. Complaint was made to the health authorities, who suspended the plumber's license; he having, besides his bad work, failed to comply with the law intended to bring every job of the kind under the notice and supervision of the health authorities, and to have it tested on completion.

SPOUTING ARTESIAN WELLS.—Iowa has a remarkable substratum of artesian water courses. The City Council of Belle Plaine have awarded the contract to control their great spouting artesian well to a Marshalltown man, who is to shut off or control the well for $2,000 per annum. The flow of the well has been 8,600,000 gallons every twenty-four hours. Since the contract for closing the old well was let, a new well, three miles southeast of town, has begun spouting, sending a two-inch stream high in the air.

BLINDNESS DUE TO DECAYED TEETH.—Dr. Widmark, a Swedish surgeon, having as a patient a young girl in whom he was unable to detect the slightest pathological changes in the right eye, but who was yet completely blind on that side, observing considerable defects in the teeth, sent her to M. Skogsborg, a dental surgeon, who found that all the upper and lower molars were completely decayed, and that in many of them the roots were inflamed. He extracted the remains of the molars on the right side, and in four days' time the sight of the right eye began to return, and on the eleventh day after the extraction of the teeth it had become normal. The diseased fangs on the other side were subsequently removed, lest they should cause a return of the opthalmic affection.—*Scien. Amer.*

A RIVAL OF PASTEUR.—A Spanish physician has lately discovered that inoculation with the virus of certain snakes is a preservative against hydrophobia in men and animals.

He has made a vast number of observations on dogs that have been bitten by vipers, and has found that they are incompetent to take the disease when afterward bitten by hydrophobic dogs. Vaccination with this virus produces little or no inconvenience, only a temporary malaise with trifling fever.

Up to the present time his experiments have proven entirely successful, and bid fair to rival in value the results obtained by Pasteur (?).—*Med. World.*

---

A timid knock at a stately door!
A thin, wan face and a liveried boor,
Good Deacon Glumm in his easy chair
Receives the message with a stare.

What! says she's hungry, much in need,
With half a dozen mouths to feed?
Well, that's a sorry case indeed,
But no concern of mine;
I didn't marry her and die,
Leaving the little ones to cry,
And for my life I don't see why
She comes round here to whine.
Give her this tract, and bid her go
To Jesus with her tale of woe,
'Tis bread, and meat, and wine."

A card! The Reverend Everso Good!
The Deacon changed to a gracious mood,
And smiling at his godly fame
That linked with charities his name,
Wrote out his check for a good round sum
To spread the "*word*" in heathendom.

